# Impact of the COVID-19 Pandemic on the Diagnosis of Colorectal Cancer within a Population-Based Organized Screening Program

**DOI:** 10.3390/cancers15194853

**Published:** 2023-10-04

**Authors:** Joaquín Cubiella, Beatriz Calderón-Cruz, Raquel Almazán, Ángel Gómez-Amorín

**Affiliations:** 1 Servicio de Aparato Digestivo, Hospital Universitario de Ourense, Grupo de Investigación en Oncología Digestiva-Ourense, Centro de Investigación Biomédica en Red Enfermedades Hepáticas y Digestivas, 32003 Ourense, Spain; 2 Unidad de Metodología y Estadística, Instituto de Investigación Sanitaria Galicia Sur, 36312 Vigo, Spain; beatriz.calderon@iisgaliciasur.es; 3 Servicio de Programas de Cribados Poblacionales, Dirección Xeral de Saude Pública, Conselleria de Sanidade, 15703 Santiago de Compostela, Spain; raquel.almazan.ortega@sergas.es (R.A.); angel.gomez.amorin@sergas.es (Á.G.-A.)

**Keywords:** colorectal cancer, COVID-19, screening, delay, adenoma

## Abstract

**Simple Summary:**

Summarize the established knowledge on this subject: (1) The COVID-19 pandemic forced the suspension of the CRC screening program and scheduled endoscopic activity. (2) The restructurion of the endoscopic unit’s organization after activity resumption limited its endoscopic capacity. (3) The delays have a presumed effect on CRC detection and the stage at diagnosis in screening programs. What are the significant and/or new findings of this study? (1) The interruption of CRC screening programs due to the COVID pandemic increased delays. (2) However, it did not reduce participation, adherence to colonoscopy, or the diagnostic yield of colonoscopy. (3) Delays within population-based CRC screening programs increase the diagnostic yield of colonoscopy without significant changes in CRC detection and its stage.

**Abstract:**

Background: The interruption of the activity of population-based organized colorectal cancer (CRC) screening programs due to the COVID pandemic may have affected their results in terms of the detection of preneoplastic lesions and CRC. We evaluated the impact of the COVID pandemic on the delays, participation, adherence to colonoscopies, lesions detected, and CRC stage at diagnosis in a CRC screening program. Methods: We analyzed all the invitations between 1 January 2019 and 31 December 2021. We defined the pandemic period as the period after 12 March 2020. We calculated the delay intervals (successive and all rounds), the rates of participation, adherence to colonoscopy after a positive fecal immunochemical test (FIT), and the diagnostic yield of colonoscopy, specifically of CRC and colorectal neoplasia (CRC and/or adenoma), as well as the CRC stage at diagnosis. Results: In the period analyzed, 976,187 invitations were sent (61.0% in the pandemic period), 439,687 FIT were returned (62.4% in the pandemic period) and 23,092 colonoscopies were performed (59.1% in the pandemic period). The colonoscopies were normal in 7378 subjects (32.4%) and CRC was detected in 916 subjects (4.0%). In successive rounds, the delay increased significantly by seven months during the pandemic period (*p* < 0.001). In all the invitations, the delay from the invitation to the colonoscopy increased significantly by 8 days (*p* < 0.001). Once adjusted for the confounding variables, the participation in the screening program increased significantly (OR = 1.1; 95% CI = 1.09–1.11), with no changes in the adherence to colonoscopy (OR = 0.9; 95% CI = 0.8–1.0). We found no differences in the diagnostic yield of colonoscopy in terms of CRC (OR = 0.90; 95% CI = 0.78–1.02) or colorectal neoplasia (OR = 0.98; 95% CI = 0.92–1.03) detection. Finally, we found no differences in the CRC stage at diagnosis (*p* = 0.2). Conclusions: Although the interruption of the CRC screening program due to the COVID pandemic increased the delays, it did not reduce participation, adherence to colonoscopy, or the diagnostic yield of colonoscopy.

## 1. Introduction

The COVID-19 pandemic forced the suspension of scheduled endoscopic activity in March 2020, including those linked to the population-based organized colorectal cancer (CRC) screening programs. The resumption of endoscopic activity was one of the greatest organizational challenges for endoscopy units. Changes were implemented in the indications and prioritization of the examinations, in the appointment and reception circuits, in the restructuring of the spaces, in the measures for the protection of the personnel, in the cleaning of the spaces, and in the control after the completion of the examinations [1]. These changes initially implied a reduction in the volume of examinations carried out and an increase in delays in the completion of these examinations. In this sense, they initially reduced the diagnoses of CRC [2] with an increase in emerging diagnoses [3]. The modeling studies carried out in the United Kingdom suggested that the delays associated with the reduction in endoscopic capacity could represent an increase of 15.3–16.6% mortality from CRC [4].

In the case of population-based CRC screening programs, the COVID-19 pandemic posed additional risk to healthy subjects invited to participate. Due to the pandemic, the activity linked to the population programs was suspended during the first wave and the resumption of the activity was heterogeneous [5]. The main risk of the prolonged suspension of the activity of the programs was the delay in the diagnosis of CRC in patients with a positive result. Delays greater than 270 days after a positive fecal immunochemical test (FIT) increased the risk of detection of CRC and advanced CRC [6].

The aim of this analysis is to describe the real impact of the temporary suspension of the CRC screening program on delays, participation, and adherence to colonoscopy as process indicators. However, our main objective is to analyze the impact on the main outcome indicators of the screening program: the detection of colorectal lesions, specifically CRC, and the stage at diagnosis, as the main surrogate of CRC-associated mortality.

## 2. Materials and Methods

### 2.1. Study Design

We performed a retrospective observational study based on the analysis of the anonymous database of the Galician CRC screening program. As of 31 December 2019, the Galician CRC screening program had a reference population of 725,254 subjects aged 50–69 years. It is based on biennial invitation to FIT (OCsensor™, Eiken Chemical Co., Tokyo, Japan) with a 20 µg/g feces threshold to refer to diagnostic colonoscopy. The information system of the screening program collects all the information related to participation, adherence to colonoscopy, delays, the colonoscopy findings, as well as the CRC stage at diagnosis. No institutional review board approval was required for this analysis as this analysis is part of the continuous quality evaluation of the screening program and the information was anonymized for it.

### 2.2. Suspension of Activity in the CRC Screening Program

The Galician CRC screening program suspended its activity on 13 March 2020. At that time, the delivery of invitation letters and fecal collectors, the appointments in primary healthcare of the subjects with a positive FIT, and the diagnostic colonoscopies were suspended. The endoscopic activity associated with the screening program was restarted in May 2020 with restrictions and the delivery of new invitations and fecal collectors was resumed completely in January 2021 [7].

### 2.3. Inclusion Criteria and Definition of the Cohorts

We included in this analysis all the invitations sent between 1 January 2019 and 31 December 2021. We defined as pre-pandemic the invitations which were sent before 13 March 2020 and as pandemic those sent after the 13 March.

### 2.4. Variables Included in the Analysis

We calculated the delay intervals according to the data included in the information system of the screening program. The intervals were defined based on the recommendations of the Spanish network of screening programs (https://cribadocancer.es/, accessed on 15 July 2023). In the successive rounds (previous return of a negative FIT) we calculated the delay from the previous round to the invitation, the FIT return, and the diagnostic colonoscopy. In all the rounds we calculated the delay from the invitation to the FIT return and to the diagnostic colonoscopy as well as the delay between the FIT return and the diagnostic colonoscopy.

We evaluated the participation rate in the screening program (number of individuals returning their FIT divided by the number of individuals invited), and the adherence to colonoscopy after a positive FIT (number of individuals undergoing colonoscopy divided by the number of individuals with a positive FIT). The findings in the colonoscopy were classified according to the European guidelines for quality assurance [8]. For this analysis, we determined the rate of CRC and colorectal neoplasia (CRC and/or adenoma) detection in patients that performed the diagnostic colonoscopy. Finally, we analyzed the CRC staging according to the AJCC classification [9].

### 2.5. Statistical Analysis

First, we performed a descriptive analysis. Quantitative variables were reported as the mean and standard deviation when they presented a normal distribution or as the median and interquartile range when the distribution was not normal. To determine normality in the variables, we used the Kolmogorov-Smirnov test. The qualitative variables were represented with their frequency and percentage. Based on the proposed objectives, we used the Chi-square test to analyze the statistical relationship between the dependent variables and the pandemic, and the Student’s *t*-test in the quantitative variables. We calculated the odds ratios (OR) and the 95% confidence interval (CI) using the Cochran–Mantel–Haenszel statistic to determine the level of association. Finally, we performed a multivariate analysis using a multivariate logistic regression model to control the potential confounding variables (round, age and sex). As a secondary analysis, we estimated the effect of the delays at pre-stablished thresholds on the diagnostic yield of colonoscopy; mainly CRC or colorectal neoplasia detection. The statistical analysis was performed using the IBM SPSS Statistics for Windows, Version 19.0, Armonk, NY, USA: IBM Corp.

## 3. Results

### 3.1. Description of the Sample

As we show in Table 1, the Galician CRC screening program sent 976,187 invitations; 61% in the pandemic period. The mean age was 58 years and 52% of the invited subjects were women. The FIT was returned by 45% of the subjects, with a positive result in 5.6%. At least one colorectal adenoma was detected in 62.7% of the 23,092 subjects that performed a colonoscopy. In addition, the colonoscopy did not detect any neoplastic findings in 32.0% of the subjects. Finally, 898 (4.0%) CRCs were detected in the analyzed period, located primarily distal to the splenic flexure in 74.4% and in a TNM stage I tumor in 44.8% of the subjects.

We evaluated the impact of the pandemic on the participation and the adherence to colonoscopy after a positive FIT, as we show in Table 1. In the multivariate logistic regression to control the potential effect of the confounding variables, we confirmed an increase in the participation (OR 1.11, 95% CI 1.09–1.116) with no changes in the adherence to colonoscopy (OR 0.93, 95% CI 0.84–1.04) during the pandemic.

### 3.2. Impact of the Pandemic on the Delays

**Figure 1:**
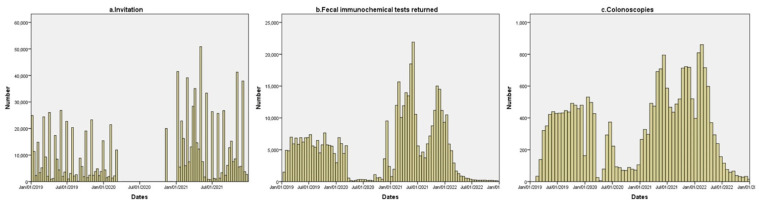
Distribution of the invitations (1 January 2019 to 31 December 2021), fecal immunochemical tests returned, and diagnostic colonoscopies.

In Figure 1 we show the distribution of the invitations, returned FITs, and colonoscopies performed during the evaluated period. As it is displayed, invitations were stopped for almost 9 months from 13 March 2020, until 11 January 2021. During the initial phase of the pandemic, a small number of FITs were returned and the diagnostic colonoscopy from pending positive results was performed.

**Figure 2:**
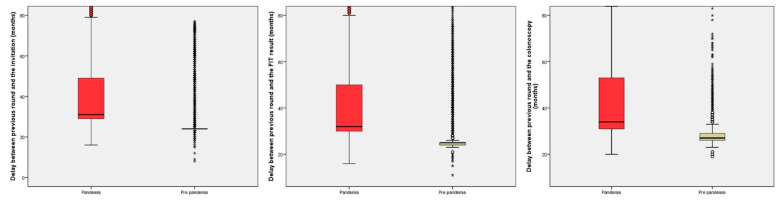
Boxplot showing the distribution of the delays in the successive rounds.

In the analysis of the delays, we found a difference of seven months in the median delay between the successive rounds in all periods evaluated, with a significant increase in the subjects with delays from the previous round of at least 27 or 30 months. The information regarding delays in the successive rounds is shown in Table 2 and Figure 2.

**Figure 3:**
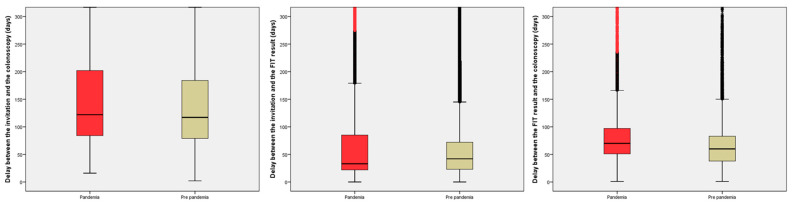
Boxplot showing the distribution of the delays after the invitation.

On the other hand, the delays after invitation are shown in Table 3 and Figure 3. To summarize the findings, the median delay from the invitation to the colonoscopy increased by 8 days, due to an increase in the delay from the positive result to the performance of the colonoscopy (10 days). In contrast, the median delay from the invitation to the FIT return was reduced by 9 days. As an example of the contradictory effect of the pandemic in the delays from the invitation, there was a significant reduction in the proportion of subjects with a delay ≥270 days from the invitation to the colonoscopy (OR 0.87, 95% CI 0.80–0.94).

### 3.3. Effect of the Pandemic on the Diagnostic Yield of Colonoscopy

As we show in Table 1, we identified statistical differences in the proportion of lesions detected in the diagnostic colonoscopies. However, in the multivariable analysis we performed to control for the confounding variables, we found no differences in the proportion of subjects with colorectal neoplasia (OR 0.97; 95% CI 0.92–1.03) or CRC (OR 0.90; 95% CI 0.78–1.02) between both periods. Finally, the multivariable analysis confirmed there were no differences in the proportion of patients with CRC TNM IV (OR 1.87; 95% CI 0.98–3.57).

### 3.4. Effect of Delays on the Diagnostic Yield of Colonoscopy

In this secondary analysis, we evaluated the impact of the delays on the diagnostic yield of colonoscopy. As we show in Table 4, the prolonged delays between the previous round and the colonoscopy did not modify the diagnostic yield of the colonoscopies. However, a delay between the previous round and the invitation of at least 30 months reduced the probability of colorectal neoplasia detection.

In addition, we also evaluated the impact of delays from invitation. As we present in Table 5, the delay from the invitation to the colonoscopy produced a statistically significant increase in colorectal neoplasia detection, with no effect on the risk of CRC detection. However, we found a contradictory effect on the delay between the FIT result and the colonoscopy. In this sense, delays longer than 90 and 180 days were associated with a statistically significant reduction of the risk of CRC (OR 0.69, 95% CI 0.58–0.82; OR 0.26, 0.16–0.44) and an increase in the risk of colorectal neoplasia (OR 1.29, 95% CI 1.20–1.37; OR 3.87, 95% CI 3.25–4.59).

## 4. Discussion

This analysis confirms that the COVID pandemic led to a significant increase in delays in population-based organized CRC screening programs. However, this increase in delays did not affect the relevant results, such as participation, adherence after a positive test, the probability of CRC detection, and its stage at diagnosis. We have only detected a consistent association between global delays and the detection of colorectal neoplasia in screening colonoscopies. On the other hand, we have only detected a statistically significant association between CRC detection and intermediate delays and with a contradictory association.

The COVID pandemic posed the greatest challenge to healthcare systems in decades. Not only did the available resources have to be dimensioned to attend to the different waves of the pandemic, but the incident pathology was at risk of not being properly attended to. In the specific case of cancer patients, the limitations of both treatment and diagnosis of incident cases were evident. In the case of CRC, two key points occurred. On the one hand, in the first wave all screening activities were suspended to reduce exposure risks both for the healthy population and for essential health services. On the other hand, when healthcare activity was reinstated, protection measures were implemented, limiting the capacity to perform colonoscopies in endoscopy units. This reduction in the diagnostic capacity of the units had a potential impact both in the evaluation of patients with symptoms and in subjects with a positive FIT in the screening programs. In this sense, the estimates posed a gloomy scenario with diagnoses at more advanced stages and an increase in mortality [2,3].

In the last twelve months, several studies have been published evaluating the real impact of the pandemic on CRC screening population programs. [10,11] Fortunately, the most pessimistic forecasts have not been confirmed. Thus, as in our study, in the recently published Dutch study, the delays associated with the pandemic have not modified the probability of detection of CRC and, only minimally, the detection of advanced neoplasia in colonoscopy. Our results are comparable to those published by the Dutch group and contradict those of other studies with smaller sample sizes where differences were determined in the participation, adherence to the screening test, and changes in the probability of detecting CRC [12,13].

We have detected a result that deserves a comment. Long delays between the positive FIT and colonoscopy were significantly associated with a reduced risk of CRC detection. It has been an unexpected result and one that is difficult to justify. Our hypothesis is that these are paucisymptomatic subjects who, after obtaining a positive result, did not wait for the screening program to schedule the diagnostic colonoscopy and, therefore, were diagnosed in a shorter interval. In contrast, those subjects without any symptoms had longer delays.

It has previously been described that increases in the delay within CRC screening programs make CRC detection more likely, especially in advanced stages [6]. Our results, as well as those obtained in the Dutch registry [11], clearly contradict this information. Although our data come from a retrospective and observational analysis, they are based on the systematic collection of population data. As in the Dutch study, the sample size is sufficient to rule out not only statistically significant but also clinically relevant differences. These results do not justify, in any case, an extension in the delays allowed by CRC screening programs. The satisfaction of the subjects involved in the population programs is key to increasing participation in them.

Our study has several strengths. We have been able to evaluate not only the process indicators of the screening program (the participation, delays in successive rounds and from the invitation, and adherence to diagnostic colonoscopies) but also the indicators of the results of the screening program: the probability of CRC and neoplasia detection in colonoscopy as well as the stage of CRC at diagnosis. The available studies have only provided partial information on each of them, focusing either on better performance or on the results. On the other hand, it is one of the few studies in which the information has been obtained from a population perspective. 

On the contrary, our study has a main limitation: we were unable to evaluate the interval CRCs [14] and, more importantly, the impact on CRC incidence in Galicia in the period of analysis, as multiple studies have already evaluated [15,16,17]. Data are only partially available in the period evaluated and will not be complete until the end of 2023 [18]. In this sense, we thought the information was relevant enough to share without the unavailable data. Additionally, due to the null impact on participation, adherence to colonoscopy and CRC detection rate, we expect the impact on interval CRC will be minimal. Finally, we could not compare the organizational particularities of the Galician CRC screening program with other screening programs. These particularities could explain some of our findings, such as the increase in the participation rate.

## 5. Conclusions

To conclude, the COVID pandemic had an impact on the Galician CRC screening program in terms of the increases in delays. However, it did not affect the relevant results: the participation in the program, adherence to diagnostic colonoscopies, detection of lesions and, above all, CRC. The pandemic has offered us a historic opportunity to understand the impact of delays on the evolution of preneoplastic lesions and CRC in asymptomatic individuals.

## Figures and Tables

**Table 1:** Baseline characteristics of the invitations in the pre-pandemic and the pandemic period.

KERRYPNX	Pre-Pandemic	Pandemic	*p*
	(*n* = 380,666)	(*n* = 595,521)	
Successive rounds (No)	93,842 (24.7%)	155,234 (26.1%)	<0.001
Gender (male)	184,064 (48.4%)	284,485 (47.8%)	<0.001
Age (years)	58.1 ± 6.0	58.5 ± 5.8	<0.001
Participation (yes)	165,321 (43.4%)	274,366 (46.1%)	<0.001
Fecal immunochemical test positive (yes)	10,046 (6.1%)	14,649 (5.3%)	<0.001
Colonoscopy (yes)	9450 (93.9%)	13,642 (93.1%)	0.01
Findings in colonoscopy			<0.001
Normal colonoscopy	3007 (31.8%)	4371 (32.0%)	
Low-risk adenoma	2232 (23.6%)	2232 (25.3%)	
Intermediate-risk adenoma	2205 (23.3%)	3162 (23.2%)	
High-risk adenoma	1533 (16.2%)	1908 (14.0%)	
Colorectal cancer	403 (4.3%)	513 (3.8%)	
Not available	70 (0.7%)	243 (1.8%)	
Colorectal cancer stage			0.3
I	187 (46.5%)	216 (43.3%)	
II	74 (18.4%)	98 (19.6%)	
III	126 (31.3%)	152 (30.5%)	
IV	14 (3.5%)	31 (6.2%)	
Unknown	1 (0.2%)	2 (0.4%)	

Qualitative variables are expressed as absolute numbers and percentages. Quantitative variables are expressed as the mean and standard deviation. Differences were analyzed with the Chi-square in the qualitative variables; and the Student’s *t*-test in the quantitative variables. Differences with *p* < 0.05 were considered statistically significant.

**Table 2:** Delays in the successive rounds according to the period analyzed.

Delays in Sucessive Rounds	Total	Pre-Pandemic	Pandemic	Significance
Delay between previous round and the invitation	*n* = 249,076	*n* = 93,842	*n* = 155,234	
Delay (months)	28 (24–34)	24 (24–24)	31 (29–49)	<0.001
Delay ≥27 months	151,715 (60.9%)	1691 (1.8%)	150,024 (96.6%)	1569 (1484–1658)
Delay ≥30 months	81,423 (32.7%)	1625 (1.7%)	79,798 (51.4%)	60.03 (57–63)
Delay between previous round and the FIT result	*n* = 223,905	*n* = 84,584	*n* = 139,321	
Delay (months)	30 (25–37)	25 (24–25)	32 (30–50)	<0.001
Delay ≥27 months	149,807 (66.9%)	11,951 (14.1%)	137,856 (98.9%)	571 (541–604)
Delay ≥30 months	94,068 (42%)	4060 (4.8%)	90,008 (64.6%)	36 (35–37)
Delay between previous round and the colonoscopy	*n* = 11,027	*n* = 4355	*n* = 6672	
Delay (months)	32 (27–40)	27 (26–29)	34 (31–53)	<0.001
Delay ≥27 months	8137 (73.8%)	1519 (34.9%)	6618 (99.2%)	228 (173–301)
Delay ≥33 months	3930 (35.6%)	327 (7.5%)	3603 (54%)	14 (12–16)
Delay ≥36 months	3048 (27.6%)	224 (5.1%)	2824 (42.3%)	13 (11–15)

Qualitative variables are expressed as absolute numbers and percentages. Quantitative variables are expressed as median and interquartile range. Differences were analyzed with the Chi-square and the Cochran–Mantel–Haenszel statistics and expressed as the odds ratio and its 95% confidence interval in the qualitative variables; and the Student’s *t*-test in the quantitative variables and expressed as the *p*-value. Differences with *p* < 0.05 were considered statistically significant. FIT, fecal immunochemical test.

**Table 3:** Delays from the invitation according to the period analyzed.

Delays from Invitation	Total	Pre-Pandemic	Pandemic	Significance
Delay between the invitation and the colonoscopy	*n* = 23,232	*n* = 9477	*n* = 13,755	
Delay (days)	121 (81–198)	116 (79–184)	124 (84–206)	<0.001
Delay ≥90 days	15,983 (68.8%)	6440 (68%)	9543 (69.4%)	1.07 (1.01–1.13)
Delay ≥180 days	6657 (28.7%)	2447 (25.8%)	4210 (30.6%)	1.27 (1.19–1.34)
Delay ≥270 days	2780 (12%)	1213 (12.8%)	1567 (11.4%)	0.87 (0.80–0.94)
Delay between the invitation and the FIT result	*n* = 43,9687	*n =* 165,321	*n* = 274,366	
Delay (days)	37 (23–79)	42 (23–72)	33 (23–85)	<0.001
Delay ≥90 days	32,097 (21.9%)	32,097 (19.4%)	64,185 (23.4%)	1.27 (1.25–1.29)
Delay ≥180 days	28,155 (6.4%)	12,650 (7.7%)	15,505 (5.7%)	0.72 (0.71–0.74)
Delay between the FIT result and the colonoscopy	*n* = 23,215	*n* = 9462	*n* = 13,753	
Delay (days)	66 (46–93)	60 (38–84)	70 (15–98)	<0.001
Delay ≥ 90 days	6156 (26.5%)	2032 (21.5%)	4124 (30%)	1.57 (1.47–1.67)
Delay ≥180 days	1473 (6.3%)	613 (6.5%)	860 (6.3%)	0.96 (0.87–1.07)

Qualitative variables are expressed as absolute numbers and percentages. Quantitative variables are expressed as median and interquartile range. Differences were analyzed with the Chi-square and the Cochran–Mantel–Haenszel statistics and expressed as the odds ratio and its 95% confidence interval in the qualitative variables; and the Student’s *t*-test in the quantitative variables and expressed as the *p*-value. Differences with *p* < 0.05 were considered statistically significant. FIT, fecal immunochemical test.

**Table 4:** Impact of the delays in the successive rounds on the diagnostic yield of colonoscopy.

Delays	Colorectal Cancer		Significance	Colorectal Neoplasia		Significance
	Yes	No		Yes	No	
	332 (3.1%)	10,552 (96.9%)		7225 (66.4%)	3659 (33.6%)	
Delay between previous round and the invitation						
≥27 months	195 (3.1%)	6144 (96.9%)	1.02 (0.18–1.27)	4172 (65.8%)	2167 (34.2%)	0.94 (0.86–1.02)
>30 months	103 (3.1%)	3198 (96.9%)	1.03 (0.81–1.31)	2134 (64.6%)	1167 (35.4%)	0.89 (0.82–0.97)
Delay between previous round and the FIT result						
≥27 months	219 (3.1%)	6959 (96.9%)	1.00 (0.79–1.25)	4734 (66.0%)	2444 (34.0%)	0.94 (0.86–1.02)
>30 months	138 (3.1%)	4355 (96.9%)	1.01 (0.81–1.26)	2919 (65.0%)	1574 (35.0%)	0.89 (0.82–0.97)
Delay between previous round and the colonoscopy						
>27 months	237 (3.0%)	7773 (97.0%)	0.89 (0.70–1.13)	5348 (66.8%)	2662 (33.2%)	1.06 (0.97–1.16)
>33 months	111 (2.9%)	3728 (97.1%)	0.91 (0.72–1.15)	2557 (66.6%)	1282 (33.4%)	1.01 (0.93–1.10)
>36 months	84 (2.8%)	2894 (97.2%)	0.89 (0.69–1.15)	1964 (66.0%)	1014 (34.0%)	0.97 (0.89–1.06)

Qualitative variables are expressed as absolute numbers and percentages. Differences were analyzed with the Chi-square and the Cochran–Mantel–Haenszel statistics and expressed as the odds ratio and its 95% confidence interval. Colorectal neoplasia: CRC or adenoma; FIT: fecal immunochemical test.

**Table 5:** Impact of the delays from the invitation on the diagnostic yield of colonoscopy.

Delay	Colorectal Cancer		Significance	Colorectal Neoplasia		Significance
	Yes	No		Yes	No	
	916 (4%)	21,847 (96%)		15,395 (67.6%)	7368 (32.4%)	
Delay between the invitation and the colonoscopy						
>90 days	645 (4.1%)	14,936 (95.9%)	1.10 (0.95–1.27)	10,656 (68.4%)	4925 (31.6%)	1.11 (1.05–1.18)
>180 days	260 (4.1%)	6099 (95.9%)	1.02 (0.88–1.18)	4584 (72.1%)	1775 (27.9%)	1.33 (1.25–1.42)
>270 days	90 (3.5%)	2474 (96.5%)	0.85 (0.68–1.06)	1917 (74.8%)	647 (25.2%)	1.47 (1.34–1.62)
Delay between the invitation and the FIT result						
>90 days	282 (5.0%)	5380 (95.0%)	1.36 (1.17–1.57)	3873 (68.4%)	1789 (31.6%)	1.04 (0.98–1.11)
>180 days	86 (4.6%)	1779 (95.4%)	1.16 (0.93–1.46)	1224 (65.6%)	641 (34.4%)	0.9 (0.82–1.0)
Delay between the FIT result and the colonoscopy						
>90 days	180 (3.1%)	5687 (96.9%)	0.69 (0.58–0.82)	4205 (71.7%)	1662 (28.3%)	1.29 (1.20–1.37)
>180 days	15 (1.2%)	1281 (98.8%)	0.26 (0.16–0.44)	1146 (88.4%)	150 (11.6%)	3.87 (3.25–4.59)

Qualitative variables are expressed as absolute numbers and percentages. Differences were analyzed with the Chi-square and the Cochran–Mantel–Haenszel statistics and expressed as the odds ratio and its 95% confidence interval. Colorectal neoplasia: CRC or adenoma. FIT, fecal immunochemical test.

## Data Availability

Data are available on demand.
